# Pulverizing medication as a harm reduction and intentional drug overdose prevention strategy: Two case studies

**DOI:** 10.1002/npr2.12189

**Published:** 2021-06-24

**Authors:** Keisuke Takanobu, Daisuke Okazaki, Shinya Watanabe, Nobuyuki Mitsui, Teruaki Tanaka

**Affiliations:** ^1^ Department of Psychiatry Hokkaido University Hospital Hokkaido Japan; ^2^ Director of the Center Hokkaido Mental Health and Welfare Center Hokkaido Japan; ^3^ Department of Psychiatry KKR Sapporo Medical Center Hokkaido Japan

**Keywords:** drug overdose, harm reduction, suicide, suicide attempted, suicide prevention

## Abstract

**Aims:**

We explored the use of pulverized medication as a new method to prevent intentional drug overdose.

**Methods:**

This case study presents data obtained from the medical records of two female patients, aged 19 and 27 years, who presented with schizophrenia and neurodevelopmental disorder, respectively. Both patients provided written informed consent. Medication was administered to the two patients in powdered form, as opposed to in tablet form, in an attempt to prevent intentional drug overdose.

**Results:**

This administration method successfully prevented intentional drug overdose for 3 and 5 years in each case, respectively. However, case‐control or prospective cohort studies are needed to rule out biases, including cognitive bias.

**Conclusion:**

Pulverizing medication is a simple and effective means of preventing intentional drug overdose by restricting access to the means of suicide, regardless of the type of mental disorder.

## Abstract 2

This case study presents two female patients, who repeated intentional drug overdose. Medication was administered to the two patients in powdered form, as opposed to in tablet form, in an attempt to prevent intentional drug overdose.

## Graphical abstract

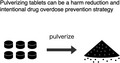

## 1 INTRODUCTION

Multiple studies have reported intentional drug overdose (IDO) as the most common form of hospital‐treated suicidal or self‐injurious behavior in Japan.^1^, ^2^ Physician‐prescribed psychotropic medications, particularly benzodiazepines, are the most frequently implicated drugs in IDO incidents requiring hospitalization.^3^ Furthermore, IDO can directly or indirectly induce fatal outcomes^4^ and is a substantial risk factor for death by suicide.^5^ While IDO directly causes death in 3%–5% of cases,^6^ it also indirectly contributes to death via other acts of self‐harm, including hanging or jumping from a height.^4^

Various approaches have been recommended to prevent IDO^7^, ^8^, ^9^; however, no decisive treatment strategy has been established. The Japanese guideline for preventing IDO focuses on discouraging doctors and other medical professionals, for example, pharmacists, from inappropriately prescribing psychotropic prescriptions.^10^ This strategy has limited value in practice, as prescribing psychotropic agents for psychiatric patients is often unavoidable.

Intentional drug overdose is often impulsive. It is also relatively easy for a patient to intentionally swallow a potentially fatal, or at least injurious, quantity of tablets in a short time. Managing psychiatric patients with a history of IDO is challenging, as the prescription of psychotropic medication is unavoidable, and the risk of IDO is high. Thus, other methods of preventing IDO in such patients are necessary.

Here, we report the cases of two patients, one with schizophrenia and one with neurodevelopmental disability, for whom we endeavored to prevent IDO by changing the dosage form from tablets to powder. Both patients described in this report provided written informed consent.

## 2 CASE PRESENTATION

### 2.1 Case 1

A 19‐year‐old woman visited our department, displaying symptoms of depressed mood, decreased motivation, suicidal ideation, and delusions. An at‐risk mental state was suspected (prodromal state of schizophrenia), and she was kept under observation. Four months later, auditory hallucinations, delusional perception, and disorganized thinking were noted. She was diagnosed with schizophrenia and was administered risperidone and other tranquilizers. She had a history of self‐injurious behavior, including wrist cutting and IDO with over‐the‐counter drugs, from the age of 14 years. On being prescribed psychotropic medication by our department, she intentionally overdosed on the medication prescribed. To reduce the IDO risk, we suggested administering a long‐acting injectable antipsychotic, which the patient refused. Given her refusal of an injectable form of the same drug, the prescribing doctor ordered that her medication (levomepromazine, 10 mg; risperidone, 5 mg; brotizolam, 0.25 mg; and zolpidem, 10 mg; interchanged over the course of treatment) be pulverized and dispensed in powder form. The prescribing doctor explained the reasons for this measure to the patient in terms of the need to reduce the risk of lethal IDO. The patient continued to attempt IDO. However, these attempts only occasionally impaired her consciousness, merely resulted in long periods of sleep, did not require emergency care, and could be managed at home. If the same medication had been in tablet form, she likely would have consumed more, with more serious consequences. When the patient reached 21 years of age, we reintroduced her medication in tablet form, permitting easier medication consumption. Three months later, she repeated IDO (levomepromazine, 5 mg × 60 tablets; risperidone, 1 mg × 10 tablets; brotizolam, 0.25 mg × 30 tablets; zolpidem, 5 mg × 60 tablets) and was transferred to our hospital. We reverted to pulverizing her medication, explaining to her the need to reduce the risk of death or significant harm. Since reintroducing her medication in powder form, no IDO has been observed for 3 years and good adherence to the prescribed medication has been confirmed.

### 2.2 Case 2

The patient presented with mild neurodevelopmental disorder (total intelligence quotient 63 on the Wechsler Adult Intelligence Scale‐Third Edition), low stress tolerance, impulsive responses to life events, and a history of self‐injurious behavior, including IDO. She visited a psychiatric clinic, where multiple drugs were prescribed off‐label for the treatment of insomnia and poor impulse control. At 27 years of age, she overdosed on valproate (200 mg × 82 tablets) and alprazolam (0.8 mg × 16 tablets) and was subsequently transported to our hospital. Her treatment was managed in the intensive care unit, and she was discharged the next day. She was refused admission to the previous psychiatric clinic due to repeated episodes of IDO and was admitted to our outpatient department. She was vulnerable to stress and unable to adequately cope with stressors in her life. Consequently, she presented with chronic suicidal ideation and frequent self‐injurious behavior, including wrist cutting and IDO. To reduce the IDO risk, the doctor ordered that her prescribed medication (brotizolam, 0.25 mg; levomepromazine, 5 mg; trazodone, 75 mg; and zolpidem, 5 mg) be pulverized and dispensed in powder form. Following discharge, the patient lived at home with her husband. Although she often exhibited impulsive self‐injurious behavior, she did not engage in the inappropriate use of prescription medications. The patient initially complained about the difficulty of taking her medication; however, this improved when she began using medical wafers to wrap the powder. She consistently adhered to her medication schedule, and at the 5‐year follow‐up, no further IDO had occurred.

In both cases described herein, the appropriate medication was prescribed by a doctor before being pulverized using a pill grinder and dispensed by a pharmacist. The powdered form of the medication was divided into portions by the pharmacist and could be taken by the patient with water.

## 3 DISCUSSION

Aside from one brief report published in Japanese,^11^ this is the first report describing the use of pulverized medication to prevent IDO in patients with schizophrenia or neurodevelopmental disorder. Our findings align with those from a previous report on patients with borderline personality disorder, in which IDO was successfully prevented by changing the form of the prescribed drugs from tablets to powder and adding a bitter taste.^12^

Pulverizing medication is useful in the treatment of psychiatric patients with high IDO risks because it “restrict[s] access to the means of suicide.”^13^ When medication is in tablet form, it is possible to swallow dozens of tablets in a short time. Conversely, powdered medications are physically difficult to consume and may cause coughing or tracheal irritation when consumed impulsively and in haste, particularly in large quantities. Even if patients attempt IDO, the resulting harm is milder due to the reduced quantity of drugs ingested, thus reducing both the direct and indirect risks of IDO, including concomitant acts of self‐harm.

There are limitations to the utility of pulverized medication. First, although it reduces the risk of IDO by restricting access to the means by which it is achieved, suicidal ideation may remain. Second, while it is difficult to swallow large powder quantities, it is not impossible for a person with strong suicidal intentions. The primary value is thus in preventing impulsive IDO, as ingesting large amounts of powdered medication requires more thought, and more time, than is typical in impulsive actions. This simple mechanism offers the patient additional time to consider their actions and can prevent impulsive attempted and completed suicides.^14^

Pulverizing medication should not be considered an intervention in isolation. Rather, it is a useful addition to the toolbox of interventions clinicians have at their disposal. It is clear that reducing polypharmacy, a common IDO prevention approach,^2^, ^3^ further reduces the IDO risk, whether medication is administered in tablet or powder form. The concurrent use of psychotherapy and pulverized medication may increase the effectiveness of both strategies. Explaining the reasons underlying pulverized medication use can serve as a learning moment as the doctor discusses the risks and mechanisms of IDO and other means of suicide with the patient. The patient must also give informed consent, understanding both the risks and benefits, at the very least in terms of the risk of deterioration in adherence and the benefit of protection from impulsive self‐harm. Such open discussions can help establish a positive therapeutic relationship between the doctor and the patient.

Even though pulverizing prescription tablets is customary in clinical practice, for patients who have difficulty swallowing tablets or who require medication via a gastric tube, it is important to note that the efficacy and safety of drugs in powdered form have not been adequately investigated.^15^ Approximately 80% of Japanese psychiatric hospitals employ pulverization for patients who need medication administered through a gastric tube, with few adverse events reported.^16^ This suggests that pulverizing tablets is a common practice in many medical institutes and can be performed safely and easily. However, pulverizing prescription tablets is mostly based on clinical empiricism. It is possible that changes in medication quality or effects (stability in light, humidity, and varying temperature; bioavailability, such as absorption; change in flavor; and drug loss during preparation) may occur due to pulverization. Despite several reports predicting adverse events resulting from crushing prescriptions analogous to pharmacological characteristics,^15^ there are currently no reports validating this concern pharmacologically. Thus, pulverization performed in accordance with dispensary guidelines or prescribing information is largely deemed safe.

The effect of pulverizing medication on medication adherence must also be considered. Even though we did not find any specific decrease in adherence in these two cases, the risk of nonadherence owing to the difficulty of medication consumption remains. Therefore, long‐acting injectable medication that reduces the risk of IDO while maintaining adherence^17^ should be considered in treatment plans whenever possible.

Confounding factors also need to be considered when interpreting the cases. In case 1, the disease state of schizophrenia may have affected the occurrence of IDO. The patient's condition was stable after the first episode, and there was no marked change in psychotic symptoms during the period. However, the minor change in medical condition may have contributed to the occurrence of IDO. In addition, there is a possibility that stress affected the occurrence of IDO. In both cases, there were occasional life events that could have caused psychological stress and triggered IDO. The changes in the frequency of IDO may have been affected by accidental life events. To eliminate the influence of these confounding factors, it is necessary to increase the number of cases and establish a control group for verification.

Our study has some limitations. First, detecting IDO outcomes is complicated by a low frequency and low rate of hospital visits.^18^ Second, the preventative effect may only be temporary; it is possible that our study's observation period was too short to detect a subsequent IDO. Third, only two cases were included, and no controls were utilized to evaluate the effectiveness of the method; thus, a case‐control or prospective cohort study is needed to clarify causality.

## 4 CONCLUSION

Pulverizing medication may be a simple and effective method of preventing IDO. Changing the drug form can restrict access to the means of suicide, regardless of the type of mental disorder. In addition to psychotherapy, judicious prescription, and limiting polypharmacy, changing the form in which medication is dispensed may be a pragmatic solution to the problem of recurrent IDO in high‐risk psychiatric patients.

## CONFLICT OF INTEREST

K. T. received personal fees from Tsumura & Co. and Otsuka Pharmaceutical outside the submitted work. N. M. received personal fees from Mochida Pharmaceutical outside the submitted work. T. T. received personal fees from Eisai, MSD, Kyowa Pharmaceutical Industry, Otsuka Pharmaceutical, Meiji Seika Pharma, Daiichi Sankyo, Dainippon Sumitomo Pharma, and Takeda Pharmaceutical outside the submitted work. The other authors declare no conflict of interest.

## AUTHOR CONTRIBUTIONS

Keisuke Takanobu performed research (mainly treated patients) and wrote the paper. Shinya Watanabe conceived of and suggested the new method. Daisuke Okazaki, Shinya Watanabe, Nobuyuki Mitsui, and Teruaki Tanaka participated in the discussion and contributed to validate the usefulness of the new method.

## INFORMED CONSENT

Informed consent was obtained from all the subjects.

## APPROVAL OF THE RESEARCH PROTOCOL BY AN INSTITUTIONAL REVIEWER BOARD

The protocol for this research project has been approved by a suitably constituted Ethics Committee of the institution, and it conforms to the provisions of the Declaration of Helsinki. Committee of Kushiro City General Hospital, Approval No. H30‐13.

## REGISTRY AND THE REGISTRATION NO. OF THE STUDY/TRIAL

Approval No. H30‐13.

## Data Availability

All the data that supports the findings of this study are available in the text of this article.
